# 

**Published:** 1865-02

**Authors:** 


Volume XXII. Number 2

### THE CHICAGO

### MEDICAL JOURNAL

DeLASKIE MILLER, M. D.,

EPHRAIM INGALS, M. D.,

Editors and Proprietors.

FEBRUARY, 1865.

CHICAGO:

J. C. W. BAILEY, BOOK AND JOB PRINTER, 130 CLARK STREET.

1865.

